# To what extent did implementing a community-embedded intervention align with the goals and roles of stakeholders in adolescent sexual and reproductive health?

**DOI:** 10.1186/s12978-024-01753-w

**Published:** 2024-02-19

**Authors:** Chinazom N. Ekwueme, Chinyere Okeke, Irene Ifeyinwa Eze, Chinyere Ojiugo Mbachu, Obinna Onwujekwe

**Affiliations:** 1 Health Policy Research Group, University of Nigeria, Enugu, Nigeria; 2 Department of Community Medicine, University of Nigeria Teaching Hospital, Enugu, Nigeria; 3 Department of Community Medicine, University of Nigeria Enugu Campus, Enugu, Nigeria; 4 Department of Community Medicine, College of Health Sciences, Alex Ekwueme Federal University Teaching Hospital, Abakaliki, Nigeria; 5 Department of Health Administration and Management, University of Nigeria Enugu Campus, Enugu, Nigeria

**Keywords:** Acceptability, Alignment, Burden, Opportunity cost, Adolescent, Sexual and reproductive health, Nigeria

## Abstract

**Background:**

Adolescents’ sexual and reproductive health (SRH) needs are largely unmet due to poor access to SRH information and services. A multicomponent community-embedded intervention, comprising advocacy to policymakers and community leaders, training of health workers on the provision of youth-friendly SRH services, and establishment of school health clubs, was implemented in Ebonyi State, Nigeria, to improve access to SRH information and services for adolescents aged 13–18 years in selected communities and secondary schools. This study explored the extent to which the intervention aligned with goals and roles of stakeholders in the State.

**Methods:**

Qualitative in-depth interviews (30) were conducted with key stakeholders in adolescent health programming in the State, and community gatekeepers (traditional and religious leaders) in the intervention communities. Sex-disaggregated focus group discussions (10) were conducted with health service providers, parents/guardians of adolescents. Data was analyzed deductively based on fit of strategy and two constructs of the Theoretical Framework for Acceptability – burden, and opportunity cost. The transcripts were coded in NVivo 12, and the subthemes that emerged from each construct were identified.

**Results:**

Stakeholders perceived the ASRH intervention activities to align with their individual goals of sense of purpose from serving the community and organizational goals of improving the visibility of adolescent reproductive health programs and aligned with their routine work. Hence, implementing or participating in the interventions was not considered a burden by many. Although the delivery of the interventions constituted additional workload and time commitment for the implementers, the benefits of partaking in the intervention were perceived to outweigh the inputs that they were required to make. Some of the community health workers in the intervention felt that provision of financial incentive will help with making the intervention less burdensome. To participate in the intervention, opportunity cost included forgoing work and business activities as well as family commitments.

**Conclusion:**

Findings from the study show that the intervention aligned with individual/organizational goals of stakeholders. To improve acceptability of the ASRH interventions, interventions should leverage on existing programs and routine work of people who will deliver the interventions.

**Supplementary Information:**

The online version contains supplementary material available at 10.1186/s12978-024-01753-w.

## Plain language summary

Adolescence is a period of transition and marked physical and mental changes with an increased need for sexual and reproductive health services. However, these needs are not usually met as adolescent face challenges in receiving care such as negative attitudes of health workers, fear of consequences of disclosing sexual and reproductive care received to parents amongst other things. An intervention aimed at improving their access to sexual and reproductive health services was implemented in Ebonyi State, Nigeria. This study assessed the alignment of the intervention to the individual and organizational goals of the stakeholders, the burden involved in participating in the intervention as well as the things needed to be forgone. Findings show that the intervention aligned with the stakeholder routine work, organizational work plans and individual goals. Participating in the intervention was not considered bothersome by many stakeholders because the impact their work make in the community makes them feel fulfilled. Participating in the intervention increases workload of the stakeholders. Stakeholders had to forgo work and business engagements to participate and some felt incentives can help to keep them motivated and interested in the project. To improve acceptability of adolescent sexual and reproductive health interventions, interventions should be tailored to the routine work of the implementers of the strategy and plans for incentives be made for stakeholders who deliver interventions.

**Supplementary Information:**

The online version contains supplementary material available at 10.1186/s12978-024-01753-w.

## Introduction

Access to sexual and reproductive health (SRH) services is key to the well-being of adolescents and essential to the achievement of one of the targets of the third sustainable development goal (SDG3), which is to achieve universal access to SRH services by 2030 [1]. Addressing adolescent sexual and reproductive health is key to reaching this target as adolescents make up 16% of the world population [2]. They also have a substantial burden of unmet SRH care needs [3, 4].

Adolescents have been shown to have relatively higher risks for sexually transmitted infections (STIs) and unwanted pregnancies [5, 6]. Globally adolescent pregnancy rate is approximately 14%, with variations across regions, and approximately 25% in sub-Saharan Africa [7]. Half of the pregnancies among adolescents in low- and middle-income countries (LMICs) are unintended, and many of these unintended pregnancies end up in unsafe abortion [8, 9]. Despite this demand for SRH care, access to SRH information and services remains limited for adolescents [10].

Even though efforts are being made to improve access to SRH information and services to adolescents in LMICs, there are still barriers to access that result from poor knowledge of SRH, judgmental attitudes of healthcare providers, little or no confidentiality while providing healthcare for them, and restrictive social norms [11–13]. Adolescents need access to appropriate SRH education to enable them to make informed choices about their SRH. Evidence has shown that provision of comprehensive sexuality education to young people improves their sexual behaviours and SRH choices [14–17].

Several intervention strategies targeted at providing sexuality education and addressing the barriers to access of SRH information among adolescents have been implemented [18–20]. However, the success of these interventions depends on various factors, such as the acceptance of the intervention and other implementation outcomes. This is particularly important in the implementation of complex health interventions which involve different components and multiple stakeholders. Proctor et al. described eight implementation outcomes – acceptability, adoption, appropriateness, feasibility, fidelity, implementation cost, penetration, and sustainability which affect the effectiveness and overall success of intervention strategies [21].

Acceptability has long been known to be an important factor for the success and long-term sustainability of interventions [22, 23]. It influences the uptake of an intervention and its continued use [24]. However, there is no consensus on its description and evaluation as several definitions exist and studies have assessed acceptability in different ways [21, 25, 26]. Acceptability has also been defined as “*a multi-faceted construct that reflects the extent to which people delivering or receiving a healthcare intervention consider it appropriate, based on anticipated or experienced cognitive and emotional responses to the intervention* [25]*”.*

Different factors can affect the acceptability of intervention by individuals involved in the delivery of intervention such as the effort and sacrifice needed to deliver intervention as well as rewards involved [27]. Many interventions are usually delivered by volunteer health workers who are usually unpaid or underpaid [28]. With their huge contribution to healthcare, there has been global debate about whether they should be paid, whether money will affect their altruistic motives and how much is enough [29–31]. Insight can be gained from evaluating acceptability from the perspectives of the stakeholders who deliver the intervention (supply side) rather than just the demand side [25].

It is therefore necessary to assess how the stakeholders accept interventions, as this will determine their willingness to continue interventions that have been proven to be effective and representative of good practices. Facilitators of intervention can then adjust interventions if acceptability is low, leading to reduced fidelity and possible lower effectiveness of the intervention.

A community-embedded intervention was implemented in Ebonyi State, Nigeria to improve access to and utilization of adolescent SRH services by adolescents aged 13–18 years. The components of the intervention were establishment of school health clubs, training of formal and informal health providers on youth-friendly SRH services, and advocacy to policy makers and community leaders.

This study aims to explore the acceptability of this multi-component, community-embedded adolescent sexual and reproductive health intervention from the perspectives of the stakeholders who participated in or benefitted from the intervention in terms of the fit of intervention strategy with their goals as well as the burden and opportunity cost of participating in the intervention. Evidence from this study will guide the design and implementation of health interventions that are more acceptable to the intervention stakeholders in a similar contextual environment.

## Methods

### Conceptual underpinning

Two theoretical frameworks were applied in the analysis: The Practical, Robust Implementation and Sustainability Model (PRISM) and the Theoretical Framework of Acceptability (TFA) [25, 32]. The concept of fit of intervention strategies with the external and internal context of intervention stakeholders from the PRISM was adapted in our study to understand how the intervention fit with the values of people involved in the intervention. The Theoretical Framework of Acceptability (TFA) is a framework which is designed to describe the properties and scope of acceptability. The framework identified seven domains of acceptability – affective attitude, burden, ethicality, intervention coherence, opportunity costs, perceived effectiveness, and self-efficacy [25]. Studies have used this framework to assess the acceptability of interventions [33, 34]. In our study, we focused on the domains of burden and opportunity cost of the TFA as those were most applicable to the objectives of the study.

### Study design and setting

We conducted a qualitative exploratory study among stakeholders of a community-embedded adolescent sexual and reproductive health (ASRH) intervention in Ebonyi State. Ebonyi State is in the southeastern part of Nigeria with three senatorial zones and 13 local government areas (LGAs); two are urban and the rest are largely rural. The State has a large youth population, with over 40 percent of the population under the age of 15 [35] and about 8.2% of 15–19 years old girls having begun child bearing [36].

The intervention was adapted from the Community-Embedded Reproductive health Care for Adolescents (CERCA) intervention implemented in Latin America [37]. It aimed to improve access to and utilization of SRH services at the primary care level for adolescents, as well as to improve SRH communication between adolescents and their parents and peers. The primary targets were in-school and out-of-school adolescents between ages 13 and 18 years, and the secondary targets were parents, teachers, community leaders, healthcare providers and policy makers. The intervention was implemented in six LGAs that were purposively selected to ensure urban–rural and geopolitical spread.

The intervention had multiple components including the establishment of school-based youth health clubs to serve as outlets for providing comprehensive sexuality education to adolescents through trained teachers or through trained young adult mentors for out-of school adolescents to build their competence in making informed SRH choices as well as refer or accompany them to seek care at intervention health facilities where necessary. Other components of the intervention were the capacity building of informal and formal healthcare providers on the provision of youth-friendly SRH services who in turn provide SRH services to the adolescents, outreach and advocacy campaigns targeted at local authorities (policy makers, community leaders and groups) and awareness campaigns through group meetings targeted at parents, adult family members and other community members to enable them interrogate existing norms and advocate for adolescent SRH in their communities. Supportive supervision was also part of the intervention with policy makers from the relevant State Ministries, local government ASRH focal persons and administrative secretaries making monthly supportive supervision visits. The intervention leveraged existing ASRH program in the State in various ways – health workers who received the training were recruited from the existing youth-friendly primary health centres in the study sites, supportive supervision of the health workers in the intervention was done by the LGA focal person for the State ASRH program.

The detailed description of the intervention can be found in an earlier published manuscript [38].

### Study participants and recruitment process

The study participants included the stakeholders involved in the facilitation of the ASRH intervention: (i) formal healthcare providers—facility health managers/officers-in-charge (OICs) of primary health centers; (ii) informal healthcare providers such as—community health workers (CHWs) and patent medicine vendors—PMVs; (iii) parents of adolescents; (iv) policy makers and program managers; (v) school teachers; an (vi) community leaders. Table 1 shows the roles of these stakeholders in the intervention. Table 2 indicates which of these stakeholders participated in the FGDs and which ones participated in the IDIs.

**Table 1:** Roles of the study participants in the ASRH intervention

Intervention stakeholder	Role in the intervention
Formal healthcare providers	Provision of adolescent-friendly SRH services to the adolescents
Informal healthcare providers	PMVs provide adolescent-friendly SRH services to adolescents and refer where necessary CHWs visit adolescents in the communities and provide SRH education. The CHWs also served as young adult mentors to the adolescents
Parents of adolescents	Participating in community group meetings
Policy makers and program managers	Supportive supervision of the provision of the implementation of school health clubs, provision of SRH services by trained health workers and community group meetings
School teachers	Facilitating school health clubs and providing SRH information through the clubs to the adolescents
Community leaders	Mobilizing community members for community group meetings

**Table 2:** Summary of FGDs and IDIs and profile of participants

Description of participants	Number of interviews	Number of participants	Gender	
			Male	Female
In-depth interviews (N = 30)				
Policymakers and partners				
State Ministry of Health	3	3	–	3
State Ministry of Education	1	1	–	1
State Ministry of Information	1	1	–	1
Legislator	1	1	1	–
State Ministry of youth and sports development	1	1	1	–
Primary Health Care Development (SPHCDA)	1	1	–	1
Media (EBBC)	2	2	1	1
SDGs	1	1	–	1
NGO	1	1	–	1
CSO	I	1	1	–
Community leaders				
Traditional rulers	2	2	2	–
Religious leader	1	1	1	–
Health service providers/supervisors and teachers				
LGA ASRH focal officers	6	6	1	5
LGA admin secs	2	2	–	2
Teachers/Principals/GC	6	6	2	4
Focus Group Discussions (10 groups)				
PHC workers (OICs)	2	7/7	7	7
PMVs	2	6/6	4	8
CHWs	2	7/6	6	7
Parents, community leaders				
Male parents	1	6	6	–
Female parents	1	6	–	6
Male community leaders	1	6	6	–
Female community leaders	1	6	–	6
Total	40	93	39	54

All the policy makers who participated in the intervention were approached to be interviewed. Other respondents were recruited purposively from those who participated in the intervention. The participants were recruited through invitation letters and scheduled appointments via phone calls. The study was explained to participants in English or the local language, if preferred, by multilingual research staff. Individual written informed consent was obtained from each participant.

### Data collection

Data was collected through 10 focus group discussions (FGDs) and 30 in-depth interviews (IDI) for a period of 1 month in 2022. The FGDs helped us to explore group shared experiences and opinions while the IDIs enabled us to explore individual experiences and perspectives. Each FGD and IDI was audio-recorded and held in-person. Each FGD and IDI was facilitated using interview guides by a pair of trained social scientists with at least two years of experience in qualitative research. No other person was present besides the participants and facilitators. The researchers were not present during the data collection. One of the facilitators took notes and observed for non-verbal cues. These notes were used to augment data analysis and interpretation. The interview guides were specifically developed for this study and the questions explored respondents’ views on how the intervention aligned to their individual and organizational goals (strategic fit), the effort needed (burden) and the things they had to forgo (opportunity cost) to implement the intervention. The interview guide had prompts to help explore all relevant aspects of the research questions. The interview guides were pilot tested in a contiguous site and modifications were made to the guide following the pretest.

FGDs for parents and adolescents were sex-disaggregated, and the number of participants per FGD was an average of six people. Each IDI lasted about 45 min to one hour, and FGD lasted between 60 and 90 min. The FGDs and IDIs were conducted in private places and convenient places for the participants at health facilities or study community.

### Data analysis

All FGDs and IDIs were transcribed verbatim and translated into English from Igbo where necessary. Data analysis of the data was done deductively using a framework approach based on fit of strategy (alignment with individual goals and organizational goals) and two domains of the Theoretical Framework of Acceptability (TFA): burden and opportunity cost [25]. The transcripts were read by all the researchers to familiarize ourselves with the data. Using TFA, the transcripts were coded with NVivo version 12 (QSR International, Victoria, Australia) independently by the researchers which was followed by group review and triangulation. The coded outputs were read iteratively to identify further sub-themes until thematic saturation was achieved. The findings were validated through a workshop where synthesized data were presented to key stakeholders. This member check confirmed our initial findings. Supplementary information using the Consolidated Criteria for Reporting Qualitative Research (COREQ) checklist [39] has been provided (See Additional file 1).

## Results

Table 2 shows a summary of the FGDs and IDIs done and the profile of the participants.

We had a total of 93 participants including 47 health service providers, 12 parents, 15 community leaders, 13 policy makers, 6 school teachers. Most of participants were female (n = 80, 56.7%).

Table 3 shows a summary of the themes and subthemes from our findings.

**Table 3:** Summary of themes and subthemes

Themes	Subthemes
Alignment with individual goals	Fit with routine work
	Preferred choice for ASRH services
	Fulfillment of the need for being an agent of positive change
Alignment with organizational goals	Visibility for ASRH programmes
Burden	Blessing rather than burden
	Ease of work
	Time
	Increased workload
Opportunity costs	Forgo work and business activities
	Forgo office engagements
	Loss of clientele
	Temporarily abandon family commitments

## Fit of strategy

### Alignment with individual goals

#### Fit with routine work

The intervention flowed into the routine work and activities of the various stakeholders who facilitated, supervised, delivered, or participated in the interventions and did not distract them from their work. They considered providing the intervention as being part of doing their routine job.

*“For us, it fits in our roles as patent medicine vendors, we are doing the work already” (P2, PMVs FGD1)*

*“I’m comfortable in doing the work, in the sense that the job did not prevent me from any other thing am doing before and it didn’t give me any discomfort at all…” (P3, PMVs FGD1 )*

#### The preferred choice for ASRH services

The intervention enabled better job satisfaction among the health providers with the intervention making their roles in the community more important with most of them being the first port of call for ASRH services.

*“It actually fits the plan, one, is that we are the first point of call in this issue of adolescent reproductive health. We, in the society, feel nothing happens; they get our consent, they come to us and discuss their problem with us, and it boosts their morale whether to continue with us or not. The way you give them what they actually need gives them an insight of either to continue or drop out” (P4, PMVs FGD2)*

The intervention made the informal health providers more popular within the community and helped them network among themselves.

*“I’m comfortable in doing the work, in the sense that the job did not prevent me from any other thing am doing before and it didn’t give me any discomfort at all, rather, it gives me the opportunity to be more popular and to establish more relationships with new friends in the community*” *(P3, PMVs FGD1)*

#### Fulfillment of the need for being an agent of positive change

For some of the stakeholders, the intervention provided opportunity to satisfy the need to be an agent of positive change in the society.

“*Being a teacher and a mother, I will say that it is in line with my schedule, I teach my pupils, and I try to use or make out time to teach them about SRH, I also discuss it with them in the class or in private, it also gives me the avenue to invite and talk to any girl whom I suspect to have a bad lifestyle, I advise them on what is good and bad, you do not need to create a special time for it since you are always with then in the class” (P2, Female Parents FGD)*

### Alignment with organizational goals

#### Visibility for ASRH programmes

Many of the stakeholders already previously involved in providing SRH services felt the interventions helped make their programmes more visible.

*“It is normal, it fits into our programmes, just that it now appears to be brighter and broadened, unlike before, it is hidden when any child is coming, she will be hiding and fearful, feeling afraid for her parents not to see her, but since you people came and introduced these interventions, they now work in freely to the health centers” (P3, OICs FGD2).*

#### Fit with program work plans

From the perspective of the health policymakers, they felt that the intervention made their roles easier as it aligned to their work plans and goals in their units and ministries. The role of supportive supervision played in the intervention package helped them in their work.

*“Every year, I will always have plans for the year, and part of my plan is to conduct retraining of the health workers, do supportive supervision, and coordinate meetings which are all core activities in your program. So it was just 100% running my programs. It was what I have planned to do that you people helped me to do, you rather strengthened my plans for the year in my unit…Sure, and in the ministry, it is called "Integrated Supportive Supervision" (IDI, Policy maker 1)*

## Burden

Participants had varied opinions about the burden of implementing the interventions. Whereas some people perceived it as non-burdensome, others felt it was burdensome. However, the most recurrent response was that it was not burdensome.

### Blessing rather than burden

Most of them reported that taking part in the intervention was not burdensome to them. They regarded it as a blessing which provided an opportunity to be of more service to the community.

*“It was not a burden to me; it was a blessing because you people now gave me the forum to do my work. So, it wasn’t a burden” (IDI, Policy maker 2)*

*“No, it's not a burden, I can say it's not a burden to me because I have taken it as an assignment I have to do for the welfare of people, I have to do it; anything I do that will benefit people is always very much interesting to me so, it's not burden.” (IDI, Traditional ruler 1)*

*“It does not affect me at all, I always create time and opportunity to meet with the village people, I’m a farmer, and I don’t have a problem meeting them, like 2 hours, I will create time to meet with the adolescents” (P5, CHWs FGD1)*

*“This school health club, I don’t see it as a burden rather, I am seeing it as a welcome development because these children, these students we are talking about our future, and if you don’t get in right now, our future is shaken but if we get it right from these our students in their different schools that are coming from some villages” (IDI, NGO)*

### Ease of work

The intervention was not seen as burdensome as it made the usual work of the facilitators easier.

*“I will tell you it was not a burden, you rather eased my work. You made it easier to me and it was in the caption of your materials like T-shirts, the caps. The motto there is “Adolescents work is task for all" so it wasn't a burden, I was ready at all times and every point in time to answer to your calls.” (IDI, Policy maker 1)*

### Time

Some people perceived participation in the intervention to be burdensome in terms of time commitment. Some of such people did not want to be seen as complaining.

*“So I don’t have any complaint but for your record, yes it cost me time, it cost me some sacrifices, off course there are some meetings” (IDI,NGO)*

Lower cadre health providers, while seeing the time commitment as a burden, felt that the provision of incentives might serve as motivation to continue the intervention.

*“It affects our time and that they should be given incentive in order to be able to continue with the program.” (P4, CHWs FGD2)*

### Increased workload

Some of the intervention implementers felt that delivering the intervention increased work demands on them which led to making having to make sacrifices to be able to implement the interventions.

*“Though the program is nice, just that in health sectors, there are always many activities and demands on us, we had to make some sacrifices to implement this program.” (P5, OICs FGD2)*

## Opportunity costs

### Forgo work and business activities

Recipients of the intervention in the communities had to forgo work and business engagements to be able to take part in the intervention. However, most did not mind as they considered the benefit of participating in the intervention to outweigh the sacrifices made.

*“On that day of the campaign, I could not go to my shop, some of my customers called to tell me that they were in the market, but I could not meet up, so they bought from other people. I gained so much knowledge on how to organize my family and community and so I did not feel so bad about what I lost that day” (P4, Female Parents FGD)*

*“During the farming season, I did not go to my farm and did not go to any of the markets where I usually go to sell my farm produce, I decided to concentrate on this because if I make any mistake in this aspect of the life of my children, it can destroy so many things for us in this community. It deprived me some of the things I am supposed to do, but it is for good” (P4, Male Parents FGD)*

*“Just like today, I had to sacrifice farm work to participate in today's discussion, and so it has been happening during the training because I discovered the benefits of getting acquainted with modern-day trends about sexual and reproductive health rights for adolescents and the need to do the needful as parents to protect their future” (P1, Male community leaders FGD)*

### Forgo office engagements

For the policymakers and facilitators of the school-based intervention component who are usually very engaged with other activities, they had to forgo conflicting tasks and activities sometimes to implement the intervention.

*“Weekly, they share topics on reproductive health and if I am selected to talk, for example, I have to set aside other things and address it. So that is the only angle I may say the program intercepts my function” (IDI, Vice Principal)*

*“Yes at that period I used to many times leave my schedule to attend to those calls, that’s one thing because I cannot be here and there. I must have to forgo one thing to do another thing.” (IDI, Policy maker3)*

### Loss of clientele

Some PMVs felt that implementing the intervention (i.e. referring their clients to the PHCs) made them lose customers to the PHCs, and in their own term, “business” as the adolescents did not return to them following referral to the PHCs.

*“Because all this one after advising them, they will not come back again and we are losing business, people in the community need to talk more about the health benefit” (P2, PMVs FGD1)*

### Temporarily abandon family commitments

Some had to forgo spending time to care for family and do family-related errands. However, most still did not regret it as they were happy with the benefits accrued from being involved with the intervention.

*“I did not get information about the campaign beforehand, I had other things I needed to attend to, my mother-in-law was hospitalized, and I am her caregiver at the hospital, due to the passion towards this intervention and campaign, I let everything go, I stayed till after 4:00 PM when the exercise ended. It denied those things from me but I have no regret because I learned what I did not know before, and I am putting them into practice”*

*“That’s the area of sacrifice when it comes to that, I think my family ooooh (she laughs) but it’s worth it, I have to give up one or two things because I remember some of the days I will come back late for my school runs, I will say it is still part of the sacrifice.”(IDI, Policymaker 4)*

## Discussion

Our findings indicate that intervention stakeholders appeared to consider the implementation of community-embedded intervention for ASRH delivered by formal and informal health providers to be generally non-burdensome. They also felt it aligned with their usual service delivery roles. The alignment of intervention interventions to their routine work and individual goals made the intervention more acceptable to the health service providers. The intervention meeting individual goals such as being more popular in the community, providing opportunity to contribute positively in the community helped the intervention stakeholders perceive it as being more acceptable to them. This finding is similar to those of results from a multi-country study done in West, Central and East Africa and studies in Tanzania which suggest that community acceptance and personal satisfaction derived from helping their communities helps improve involvement of health workers in implementing interventions [40–42]. From the multi-country study involving Nigeria, Cameroon and Uganda, health workers involved in community-based interventions, were happy with the sense of personal purpose being part of the intervention that led to a reduction in disease prevalence gave them [40]. Lay health workers in Tanzania continued to provide services because of the personal pride they derived from serving the community [41, 42]. This reinforces the need to design intervention strategies that align with the roles of the implementers and stakeholders as this may lead to positive outcomes.

Policy makers in our study considered the ASRH intervention activities aligned with their program objectives and work plans. This is important in designing complex health interventions to ensure acceptability by policy-makers and increase the chances of scaling up the intervention strategies into policy. Studies have shown that low political will and poor support from policymakers and local stakeholders are barriers to the use of health interventions and their sustainment [43]. This can be improved by aligning interventions to fit identified policy priorities in a particular setting.

Although implementing the intervention created more work for the stakeholders delivering the intervention, they did not consider it a burden as the ASRH interventions provided opportunities to be of service to the community and also to learn more skills. However, a study done in Zambia showed that teachers who facilitated youth clubs providing comprehensive sexuality education felt that the additional workload was a burden as they had to find time to go through the training manuals and prepare for facilitating the youth club in addition to preparing for their usual classes [44]. To improve the acceptability of interventions among stakeholders who will facilitate them, the interventions should be packaged in a way to reduce the burden of implementing the interventions on implementing stakeholders. 

Community members had to forgo work and business engagements as well as time for family commitments to participate in the intervention, but they felt that the benefits of participating in the intervention made up for it. Policy makers and school facilitators had to forgo conflicting tasks at work to implement the intervention. In contrast, teaching commitments prevented teachers from facilitating school-based components of an intervention target at improving youth health outcomes in a study at India [45]. Our study suggests that the benefits from involvement in the intervention cover up for the opportunity costs. However, findings from studies suggest that depending on notions of altruism among intervention facilitators in resource-limited settings is unsustainable [46, 47].

Despite general acceptability, our findings suggest that a token of financial incentive to lower cadre health workers like the CHWs could have been a source of added motivation to deliver the intervention. This conforms to findings from other studies in similar settings where CHWs and lay health workers who implemented health intervention expressed a desire for provision of financial incentives to improve motivation and make up for the burden of delivering the intervention [48–51]. Allowance for adequate financial compensation in the design of interventions for health workers and lay workers involved in the delivery would help improve acceptability and improve the outcome of the intervention.

### Study limitation

While our study collected data from various stakeholders and thus provides perspectives of burden and opportunity cost of implementing ASRH intervention from different kinds of stakeholders, a mixed-method approach may have helped to determine the magnitude of the various issues surrounding burden and opportunity cost of this ASRH intervention in those communities.

## Conclusion

This study provides insight into the fit of strategy, burden, and opportunity cost of delivering an ASRH intervention from the perspectives of the intervention stakeholders. Findings from this study can guide the design of health interventions to make them more acceptable to intervention stakeholders by ensuring that the intervention strategies fit the individual and organizational goals of both the recipients and the implementers.

It is important to address issues of incentives and compensation for the burden and opportunity cost of delivering the interventions as expecting intervention facilitators to sustain involvement in interventions because it is a “blessing” and provides opportunity to serve may not make it sustainable and may be exploitative.

### Supplementary Information

**Additional file 1. **Consolidated criteria for reporting qualitative studies (COREQ) checklist.

## Data Availability

The datasets used and/or analysed during the current study are available from the corresponding author on reasonable request.

## Abbreviations

SRH: Sexual and reproductive health
ASRH: Adolescent sexual and reproductive health
SDG: Sustainable development goal
STI: Sexually transmitted infection
LMICs: Low and middle-income countries
TFA: Theoretical framework of acceptability
LGAs: Local government areas
OICs: Officers-in-charge
CHWs: Community health workers
PMVs: Patent medicine vendors
FGD: Focus group discussion
IDI: In-depth interview

## References

[1] SDGS. Goal 3 | Department of Economic and Social Affairs. United Nations Sustainable Development Goals (UNSDGS). 2015. https://sdgs.un.org/goals/goal3. Accessed 24 Sep 2022.

[2] UNICEF. Adolescents Statistics - UNICEF DATA [Internet]. Adolescents Overview. 2019. https://data.unicef.org/topic/adolescents/overview/. Accessed 02 Feb 2023.

[3] Morris JL, Rushwan H (2015). Adolescent sexual and reproductive health: the global challenges. Int J Gynecol Obstet.

[4] Sadinsky S, Nuñez AJ, Nabulega S, Riley T, Ahmed Z, Sully E. From bad to worse: the COVID-19 pandemic risks further undermining adolescents’ sexual and reproductive health and rights in many countries. Guttmacher Inst. 2020. https://www.guttmacher.org/article/2020/08/bad-worse-covid-19-pandemic-risks-further-undermining-adolescentssexual-and. Accessed 02 Feb 2023.

[5] Scott ME, Wildsmith E, Welti K, Ryan S, Schelar E, Steward-Streng NR (2011). Risky adolescent sexual behaviors and reproductive health in young adulthood. Perspect Sex Reprod Health.

[6] Epstein M, Bailey JA, Manhart LE, Hill KG, Hawkins JD, Haggerty KP (2014). Understanding the link between early sexual initiation and later sexually transmitted infection: test and replication in two longitudinal studies. J Adolesc Health.

[7] Early childbearing and teenage pregnancy rates by country - UNICEF DATA. https://data.unicef.org/topic/child-health/adolescent-health/. Accessed 02 Feb 2023.

[8] Sully EA, Biddlecom A, Darroch JE, Riley T, Ashford LS, Lince-Deroche N, et al. Adding it up: investing in sexual and reproductive health 2019. 2020 Jul 28. https://www.guttmacher.org/report/adding-it-up-investing-in-sexual-reproductive-health-2019. Accessed 02 Feb 2023.

[9] Bankole A, Remez L, Owolabi O, Philbin J, Williams P. From Unsafe to Safe Abortion in Sub-Saharan Africa: Slow but Steady Progress. Guttmacher Institute. 2020. https://www.guttmacher.org/report/from-unsafe-to-safe-abortion-in-subsaharan-africa. Accessed 02 Feb 2023.

[10] Chandra-Mouli V, Svanemyr J, Amin A, Fogstad H, Say L, Girard F (2015). Twenty years after international conference on population and development: where are we with adolescent sexual and reproductive health and rights?. J Adolesc Heal.

[11] Chandra-Mouli V, McCarraher DR, Phillips SJ, Williamson NE, Hainsworth G (2014). Contraception for adolescents in low and middle income countries: needs, barriers, and access. Reprod Health.

[12] Kennedy EC, Bulu S, Harris J, Humphreys D, Malverus J, Gray NJ (2013). “Be kind to young people so they feel at home”: a qualitative study of adolescents’ and service providers’ perceptions of youth-friendly sexual and reproductive health services in Vanuatu. BMC Health Serv Res.

[13] Thongmixay S, Essink DR, De Greeuw T, Vongxay V, Sychareun V, Broerse JEW (2019). Perceived barriers in accessing sexual and reproductive health services for youth in Lao People’s Democratic Republic. PLoS ONE.

[14] Kirby DB, Laris BA, Rolleri LA (2007). Sex and HIV education programs: their impact on sexual behaviors of young people throughout the world. J Adolesc Health.

[15] Kohler PK, Manhart LE, Lafferty WE (2008). Abstinence-only and comprehensive sex education and the initiation of sexual activity and teen pregnancy. J Adolesc Heal.

[16] Esere MO (2008). Effect of sex education programme on at-risk sexual behaviour of school-going adolescents in Ilorin, Nigeria. Afr Health Sci.

[17] Reis M, Ramiro L, de Matos MG, Diniz JA (2011). The effects of sex education in promoting sexual and reproductive health in Portuguese university students. Procedia - Soc Behav Sci.

[18] Obiezu-Umeh C, Nwaozuru U, Mason S, Gbaja-Biamila T, Oladele D, Ezechi O (2021). Implementation strategies to enhance youth-friendly sexual and reproductive health services in Sub-Saharan Africa: a systematic review. Front Reprod Heal.

[19] Meherali S, Rehmani M, Ali S, Lassi ZS (2021). Interventions and strategies to improve sexual and reproductive health outcomes among adolescents living in low- and middle-income countries: a systematic review and meta-analysis. Adolescents.

[20] Desrosiers A, Betancourt T, Kergoat Y, Servilli C, Say L, Kobeissi L (2020). A systematic review of sexual and reproductive health interventions for young people in humanitarian and lower-and-middle-income country settings. BMC Public Health.

[21] Proctor E, Silmere H, Raghavan R, Hovmand P, Aarons G, Bunger A (2011). Outcomes for implementation research: conceptual distinctions, measurement challenges, and research agenda. Adm Policy Ment Health.

[22] Davis FD (1993). User acceptance of information technology: system characteristics, user perceptions and behavioral impacts. Int J Man Mach Stud.

[23] Kazdin AE, Wilson GT (1978). Criteria for evaluating psychotherapy. Arch Gen Psychiatry.

[24] Sekhon M, Cartwright M, Lawes-Wickwar S, McBain H, Ezra D, Newman S (2021). Does prospective acceptability of an intervention influence refusal to participate in a randomised controlled trial? An interview study. Contemp Clin trials Commun.

[25] Sekhon M, Cartwright M, Francis JJ (2017). Acceptability of healthcare interventions: an overview of reviews and development of a theoretical framework. BMC Health Serv Res.

[26] Acceptability Instruments | Implementation outcomes. https://implementationoutcomerepository.org/implementation-outcomes/acceptability-instruments. Accessed 03 Feb 2023.

[27] Pallas SW, Minhas D, Pérez-Escamilla R, Taylor L, Curry L, Bradley EH (2013). Community health workers in low-and middle-income countries: what do we know about scaling up and sustainability?. Am J Public Health.

[28] Ballard M, Westgate C, Alban R, Choudhury N, Adamjee R, Schwarz R (2021). Compensation models for community health workers: comparison of legal frameworks across five countries. J Glob Health.

[29] Bhattacharyya K, Winch P, LeBan K, Tien M (2001). Community health worker incentives and disincentives: how they affect motivation, retention, and sustainability.

[30] Maes K (2015). “Volunteers are not paid because they are priceless”: community health worker capacities and values in an AIDS treatment intervention in urban Ethiopia. Med Anthropol Q.

[31] Kironde S, Bajunirwe F (2002). Lay workers in directly observed treatment (DOT) programmes for tuberculosis in high burden settings: should they be paid? A review of behavioural perspectives. Afr Health Sci.

[32] Feldstein AC, Glasgow RE (2008). A practical, robust implementation and sustainability model (PRISM) for integrating research findings into practice. Jt Comm J Qual patient Saf.

[33] Murphy AL, Gardner DM (2019). Pilot testing the theoretical framework of acceptability in a process evaluation of a community pharmacy-based men’s mental health promotion program. SAGE Open.

[34] Timm L, Annerstedt KS, Ahlgren JÁ, Absetz P, Alvesson HM, Forsberg BC (2022). Application of the theoretical framework of acceptability to assess a telephone-facilitated health coaching intervention for the prevention and management of type 2 diabetes. PLoS ONE.

[35] Health Policy Plus. Nigeria Population and Development: Ebonyi State. FACTSHEET Sept 2017 Popul. 2017; 1–2. http://www.healthpolicyplus.com/ns/pubs/7149-7286_EbonyiRAPIDFactSheet.pdf. Accessed 03 Feb 2023.

[36] National Population Commission (NPC) [Nigeria], ICF. Nigeria Demographic Health Survey 2018. DHS Progr ICF Rockville, Maryland, USA. 2019; 748. https://dhsprogram.com/publications/publication-fr359-dhs-final-reports.cfm. Accessed 03 Feb 2023.

[37] Decat P, Nelson E, De Meyer S, Jaruseviciene L, Orozco M, Segura Z (2013). Community embedded reproductive health interventions for adolescents in Latin America: development and evaluation of a complex multi-centre intervention. BMC Public Health.

[38] Mbachu CO, Clara Agu I, Onwujekwe O (2020). Collaborating to co-produce strategies for delivering adolescent sexual and reproductive health interventions: processes and experiences from an implementation research project in Nigeria. Health Policy Plan.

[39] Tong A, Sainsbury P, Craig J (2007). Consolidated criteria for reporting qualitative research (COREQ): a 32-item checklist for interviews and focus groups. Int J Qual Heal Care.

[40] World Health Organization. Community-directed interventions for major health problems in Africa: a multicountry study final report. Special Programme for research and training in tropical diseases. Geneva. 2008. https://tdr.who.int/publications/m/item/2008-05-09-community-directed-interventions-for-major-health-problems-in-africa. Accessed 03 Feb 2023.

[41] Ahluwalia IB, Schmid T, Kouletio M, Kanenda O (2003). An evaluation of a community-based approach to safe motherhood in northwestern Tanzania. Int J Gynaecol Obstet.

[42] Greenspan JA, McMahon SA, Chebet JJ, Mpunga M, Urassa DP, Winch PJ (2013). Sources of community health worker motivation: a qualitative study in Morogoro Region, Tanzania. Hum Resour Health.

[43] Yamey G (2012). What are the barriers to scaling up health interventions in low and middle income countries? A qualitative study of academic leaders in implementation science. Global Health.

[44] Chirwa-Kambole E, Svanemyr J, Sandøy I, Hangoma P, Zulu JM (2020). Acceptability of youth clubs focusing on comprehensive sexual and reproductive health education in rural Zambian schools: a case of Central Province. BMC Health Serv Res.

[45] Balaji M, Andrews T, Andrew G, Patel V (2011). The acceptability, feasibility, and effectiveness of a population-based intervention to promote youth health: an exploratory study in Goa, India. J Adolesc Heal.

[46] Akintola O (2011). What motivates people to volunteer? The case of volunteer AIDS caregivers in faith-based organizations in KwaZulu-Natal. South Africa. Health Policy Plan.

[47] Maes K (2012). Volunteerism or labor exploitation? Harnessing the volunteer spirit to sustain AIDS Treatment Programs in Urban Ethiopia. Hum Organ.

[48] Cataldo F, Kielmann K, Kielmann T, Mburu G, Musheke M (2015). ‘Deep down in their heart, they wish they could be given some incentives’: a qualitative study on the changing roles and relations of care among home-based caregivers in Zambia. BMC Health Serv Res.

[49] Odiachi A, Al-Mujtaba M, Torbunde N, Erekaha S, Afe AJ, Adejuyigbe E (2021). Acceptability of mentor mother peer support for women living with HIV in North-Central Nigeria: a qualitative study. BMC Pregnancy Childbirth.

[50] Jigssa HA, Desta BF, Tilahun HA, McCutcheon J, Berman P (2018). Factors contributing to motivation of volunteer community health workers in Ethiopia: the case of four woredas (districts) in Oromia and Tigray regions. Hum Resour Health.

[51] Sam-Agudu NA, Odiachi A, Bathnna MJ, Ekwueme CN, Nwanne G, Iwu EN (2018). “They do not see us as one of them”: a qualitative exploration of mentor mothers’ working relationships with healthcare workers in rural North-Central Nigeria. Hum Resour Health.

