# Supplementary material for: To what extent did implementing a community-embedded intervention align with the goals and roles of stakeholders in adolescent sexual and reproductive health?
Source: Reprod Health. 2024 Feb 19;21:27. doi: 10.1186/s12978-024-01753-w (PMC10877846; doi:10.1186/s12978-024-01753-w)
Supplement: Supplementary file 1 — Additional file 1. Consolidated criteria for reporting qualitative studies (COREQ) checklist. [file 12978_2024_1753_MOESM1_ESM.docx]

**Consolidated criteria for reporting qualitative studies (COREQ) checklist for manuscript titled:**

***To what extent did implementing a community-embedded intervention align with the goals and roles of stakeholders in adolescent sexual and reproductive health?***

| **No.** | **Item** | **Guide Questions/Description** | **Response (and Location of Relevant Narrative in Manuscript where applicable)** |
| --- | --- | --- | --- |
| **Domain 1: Research Team and Reflexivity** | | | |
| Personal Characteristics | | | |
| 1 | Interviewer/facilitator | Which author/s conducted the interview or focus group? | None of the researchers conducted the focus group discussions and In-depth interviews. FGDs and IDIs were conducted by research assistants who were social scientists (Methods – Data collection) |
| 2 | Credentials | What were the researcher’s credentials? E.g. PhD, MD | CNE (MBBS), CO (MBBS, MPH), IE (MBBS, MPH), COM (MBBS, MPH) and OO(MBBS, PhD) |
| 3 | Occupation | What was their occupation at the time of the study? | CNE, Senior Registrar, Department of Community Medicine and Public Health and Research Associate, CO, Senior Lecturer, Community Medicine/Public Health and Honorary Consultant, Research Associate  IE, Senior Lecturer, Community Medicine/Public Health and Honorary Consultant, Research Associate  COM, Public health, health systems and policy Professor, Honorary consultant  OO, Professor of health systems and policy, senior author |
| 4 | Gender | Was the researcher male or female? | Female researchers: CNE, CO, IE, COM. Male researcher: OO. |
| 5 | Experience and training | What experience or training did the researcher have? | CNE (Trained, 4 years of qualitative research experience)  CO (Trained; over 8 years of qualitative research experience)  IE (Trained; over 8 years of qualitative research experience)  COM(Trained; over 12 years of qualitative research experience)  OO (Trained; over 20 years of qualitative research experience |
| Relationship with participants | | | |
| 6 | Relationship established | Was a relationship established prior to study commencement? | Prior to the project commencement, relationship was established with some of the study participants during a stakeholder engagement workshop done to engage and get the buy-in of the stakeholders |
| 7 | Participant knowledge of the interviewer | What did the participants know about the researcher? e.g. personal goals, reasons for doing the research | During the workshop, researchers introduced themselves stating where they worked (with a research group/university and not for the health facility or government), and reasons for doing the research, namely, to improve the access of adolescents to sexual and reproductive health services. During consent process, the research assistants introduced the research group and objective of the study |
| 8 | Interviewer characteristics | What characteristics were reported about the interviewer/facilitator? Eg Bias, assumptions, reasons and interests in the research topic | CNE is a female public health physician interested in efficient health systems delivery for maternal, child and adolescent health.  CO is a female public health physician and research interests in health systems research and policy analysis  IE female public health physician and research interests in health systems research and policy analysis  COM is a female public health physician and health systems researcher interested in improving adolescent and young people’s sexual and reproductive health with special interest in gender issues and gender transformative approaches and has been doing research in the study setting for about 5 years now  OO is a male medically trained health economist and health system and policy researcher of international repute  All authors are Nigerians and Igbo, same ethnic group as the dominant group of the study setting, and understand the cultural and religious context of the study setting |
| **Domain 2: Study Design** | | | |
| Theoretical Framework | | | |
| 9 | Methodological orientation and Theory | What methodological orientation was stated to underpin the study? e.g. grounded theory, discourse analysis, ethnography, phenomenology, content analysis | Inductive methodology with framework analysis. (Methods- Data analysis) |
| Participant selection | | | |
| 10 | Sampling | How were participants selected? e.g. purposive, convenience, consecutive, snowball | All policymakers were interviewed. Purposive sampling for other participants. (Methods- Study participants and recruitment process) |
| 11 | Method of approach | How were participants approached? e.g. face-to-face, telephone, mail, email | Through invitations letters and phone calls. (Methods- Study participants and recruitment process) |
| 12 | Sample size | How many participants were in the study? | A total of 141 participants in 18 FGDs and 30 IDIs (Results and Table 1. Summary of FGDs and IDIs and profile of participants) |
| 13 | Non-participation | How many people refused to participate or dropped out? Reasons? | Four people could not come for the FGD. Two of them was due to conflicting schedule. The for no-show for the other two was not known |
| Setting | | | |
| 14 | Setting of data collection | Where was the data collected? e.g. home, clinic, workplace | The FGDs and IDIs were conducted in private spaces at health facilities or in the study community. (Methods- Data collection) |
| 15 | Presence of non-participants | Was anyone else present besides the participants and researchers? | No one else was present besides participants and pair of facilitators. (Methods-Data collection) |
| 16 | Description of sample | What are the important characteristics of the sample? e.g. demographic data, date | Most study participants were female. (Results). |
| Data collection | | | |
| 17 | Interview guide | Were questions, prompts, guides provided by the authors? Was it pilot tested? | The interviewers used FGD and IDI guides to collect data. The guides were pilot-tested. Participants who took part in the pilot test did not participate in the data collection. (Methods- data collection) |
| 18 | Repeat interviews | Were repeat interviews carried out? If yes, how many? | No, repeat interviews of participants were not conducted. |
| 19 | Audio/visual recording | Did the research use audio or visual recording to collect the data? | FGDs and IDIs were audio-recorded and later transcribed. There were no visual recordings. (Methods- Data collection). |
| 20 | Field notes | Were field notes made during and/or after the interview or focus group? | Yes, an observer made field notes during the FGDs to augment data analysis and interpretation. (Methods-Data collection) |
| 21 | Duration | What was the duration of the interviews or focus group? | Each FGD lasted between 60 and 90 minutes, and each IDI took approximately 45 minutes. (Methods- Data Collection) |
| 22 | Data saturation | Was data saturation discussed? | Thematic saturation was discussed and reached in the course of the FGDs and IDIs. (Methods- Data analysis). |
| 23 | Transcripts returned | Were transcripts returned to participants for comment and/or correction? | No. Results were shared with some of the participants at a data validation workshop (Methods – Data analysis) |
| **Domain 3: Analysis and Findings** | | | |
| Data analysis | | | |
| 24 | Number of data coders | How many data coders coded the data? | Coding was performed independently by all the researchers - CNE, CO, IE, COM, OO  (Methods-Data analysis) |
| 25 | Description of the coding tree | Did authors provide a description of the coding tree? | In initial coding, fit of strategy, burden and opportunity cost from the Theoretical Framework of Acceptability was use (Methods- Data analysis) |
| 26 | Derivation of themes | Were themes identified in advance or derived from the data? | Themes were identified in advance using the deductive approach (Methods- Data Transcription and Analysis) |
| 27 | Software | What software, if applicable, was used to manage the data? | Nvivo qualitative software was used to manage the data and analyze the data  (Methods- Data analysis) |
| 28 | Participant checking | Did participants provide feedback on the findings? | Member check was done via a data validation workshop with stakeholders where results were presented to them for their input. Stakeholders provided feedback and confirmed our findings. (Methods- Data analysis) |
| Reporting | | | |
| 29 | Quotations presented | Were participant quotations presented to illustrate the themes / findings? Was each quotation identified? e.g. participant number | Participant quotations are presented and identified by participant group and number (Throughout Results section) |
| 30 | Data and findings consistent | Was there consistency between the data presented and the findings? | Yes, discussion on findings was written to align with the results/data presented |
| 31 | Clarity of major themes | Were major themes clearly presented in the findings? | Major themes are clearly presented in the Results section and in Table 2. |
| 32 | Clarity of minor themes | Is there a description of diverse cases or discussion of minor themes? | Yes, minor themes and/or divergent cases were discussed under each of the major themes (Results) |
